# Thermochemical and Kinetic Investigation of CH_3_NH_2_ Production in Titan’s Atmosphere

**DOI:** 10.1021/acsomega.5c09060

**Published:** 2025-11-17

**Authors:** Ghuerda L. Mayr, Isabela S. Vieira, Rene F. K. Spada

**Affiliations:** † Departamento de Física, 74360 Instituto Tecnológico de Aeronáutica, São José dos Campos, São Paulo 12228-900, Brasil; ‡ Laboratório de Computação Científica Avançada e Modelamento (Lab-CCAM), Instituto Tecnológico de Aeronáutica, São José dos Campos, São Paulo 12228-900, SP, Brazil

## Abstract

The atmosphere of Titan is predominantly composed of nitrogen-, methane-, and carbon-based compounds. When exposed to energy sources such as solar radiation, these species can undergo complex chemical transformations, leading to the formation of organic molecules, such as methylamine, as well as its photodissociation. The present study aims to investigate the thermochemical and kinetic properties of elementary reactions involved in the production of methylamine (CH_3_NH_2_) over a temperature range of 75 to 300 K employing reliable electronic structure and chemical kinetics methods. The first reaction pathway (R_a_) initiates with the reactants CH_2_ and NH_2_, yielding CH_2_NH_2_ (CH_2_ + NH_2_ → CH_2_NH_2_, *R*_a1_) without an activation barrier. The formed CH_2_NH_2_ can further react with NH, leading to the formation of CH_3_NH_2_ and N (CH_2_NH_2_ + NH → CH_3_NH_2_ + N, *R*_a2_), if it overcomes a barrier of 25.5 kJ·mol^–1^. The second reaction pathway (R_b_) involves the reactants CH_3_ and NH, producing CH_3_NH (CH_3_ + NH → CH_3_NH, *R*_b1_), also without an activation barrier. This species can later react with NH to produce CH_3_NH_2_ and N (CH_3_NH + NH → CH_3_NH_2_ + N, *R*_b2_), as long as they overcome an energy barrier of 16.1 kJ·mol^–1^. The rate coefficients calculated at 100 K are 3.6 × 10^–9^ and 2.5 × 10^–18^ cm^3^·molecule^–1^·s^–1^ for the consecutive reactions *R*_a1_ and *R*_a2_, respectively, and 3.8 × 10^–9^ and 6.5 × 10^–17^ cm^3^·molecule^–1^·s^–1^ for the consecutive reactions *R*_b1_ and *R*_b2_, respectively. Our results contribute to the modeling of Titan’s atmosphere (and such environments), enabling the production of the CH_2_NH_2_ and CH_3_NH radicals that may participate in other reactions for the production of more complex hydrocarbons and nitrogen compounds, as well as an alternative feasible mechanism for the production of methylamine.

## Graphical abstract

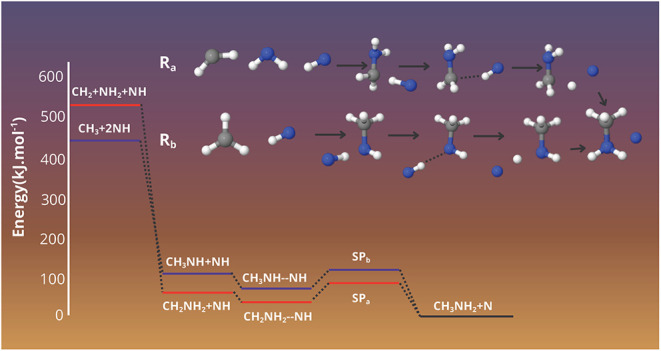

## 1 Introduction

Methylamine was first detected in the interstellar medium toward the molecular clouds Sagittarius B2 and Orion A.^1^ It can be found and its existence is suggested in various regions of the interstellar medium, including molecular clouds,^2,3^ nebulae,^4,5^ interstellar ices,^6^ novae,^7^ planetary atmospheres such as Jupiter’s^8,9^ and Saturn’s moons Enceladus^10^ and Titan,^11–14^ as well as rocky exoplanets.^15^ It is also present in comets^16,17^ and can be formed in protostellar environments,^18^ where it plays a role in the synthesis of interstellar glycine.^19,20^ As a precursor in the formation of proteinogenic amino acids, its identification in the interstellar medium contributes to a better understanding of the formation of complex organic molecules.^21^

In this context, Titan, Saturn’s largest moon, stands out for its rich atmospheric chemistry involving nitrogen, carbon, and hydrogen compounds.^22^ Data obtained by Voyager 1 in 1980 clearly demonstrated that Titan’s atmosphere is primarily composed of nitrogen, with a smaller amount of methane, as well as the presence of argon (Ar), hydrogen (H_2_), hydrogen cyanide (HCN), and several hydrocarbons, including acetylene (C_2_H_2_), ethylene (C_2_H_4_), and ethane (C_2_H_6_).^23^ Moreover, it is noteworthy that Torokova et al. experimentally simulated Titan’s atmosphere using methane and molecular nitrogen and observed the formation of methylamine.^24^

Krasnopolsky,^11^ using data from the Cassini-Huygens probe, simulated this atmosphere, considering the photodissociation of the methylamine molecule (CH_3_NH_2_) into CH_3_ and NH_2_, taking values reported by Hubin-Franskin et al.,^25^ as well as the reverse process in the presence of a third inert body, both processes described by the following reactions

R1 $${\mathrm{CH}}_{3}{\mathrm{NH}}_{2}+\left(h\nu \right)\to {\mathrm{CH}}_{3}+{\mathrm{NH}}_{2}$$

R2 $${\mathrm{NH}}_{2}+{\mathrm{CH}}_{3}+M\to {\mathrm{CH}}_{3}{\mathrm{NH}}_{2}+M$$

However, alternative pathways for the decomposition and production of this molecule were not considered.

The reaction of methylamine has been studied with various other molecules, including O_2_,^26^ OH,^27–33^ CN,^34,35^ O_3_,^36^ Cl,^37^ CO,^38^ CH,^39^ and HS^+^,^40^ as well as its synthesis from CO_2_, H_2_, and NH_3_.^41^ In particular, the hydrogen-assisted decomposition of CH_3_NH_2_ has been studied by Kerkeni and Clary,^42^ and Zhang et al.^43^

In this context, with the aim of improving such investigations of Titan’s atmosphere and other environments in which methylamine may play a role, a thermochemical and kinetic study was conducted on the following reactions that can lead to the production and decomposition of CH_3_NH_2_, through the following elementary steps,

Ra $$\begin{array}{l} {\mathrm{CH}}_{2}+{\mathrm{NH}}_{2}+M\overset{R_{a1}}{\to }{\mathrm{CH}}_{2}{\mathrm{NH}}_{2}+M\\ {\mathrm{CH}}_{2}{\mathrm{NH}}_{2}+\mathrm{NH}\overset{R_{a2}}{\underset{R_{a3}}{⇌}}{\mathrm{CH}}_{3}{\mathrm{NH}}_{2}+N \end{array}$$

Rb $$\begin{array}{l} {\mathrm{CH}}_{3}+\mathrm{NH}+M\overset{R_{b1}}{\to }{\mathrm{CH}}_{3}\mathrm{NH}+M\\ {\mathrm{CH}}_{3}\mathrm{NH}+\mathrm{NH}\overset{R_{b2}}{\underset{R_{b3}}{⇌}}{\mathrm{CH}}_{3}{\mathrm{NH}}_{2}+N \end{array}$$

where *R*_a1_ and *R*_b1_ occur on a duplet surface, while *R*_a2_ and *R*_b2_ take place in a quartet surface. The symbol *M* represents an inert body required to dissipate the energy from the products of *R*_a1_ and *R*_b1_ by collision; otherwise, the products might retain enough energy to redissociate into reactants. Hence, our results are valid for the high-pressure limit. We follow the statement by Nixon^44^ that Titan’s atmosphere, especially the low-atmosphere with its pressure of approximately 1.5 bar, is dense enough to ensure that the collision rate among molecules is high enough to stabilize the reaction products by collisions effectively, preventing their redissociation.

Also, according to Nixon,^44^ all initial species (CH_3_, CH_2_, NH_2_, and NH) already play a role in the chemistry of Titan’s atmosphere. First, considering carbon-based compounds, CH_3_ and CH_2_ can be produced in that atmosphere by photolysis of methane (CH_4_) in the atmosphere, and these radicals are involved in a chain of reactions to form the other molecules already found in Titan’s atmosphere, including hydrocarbons and nitrogen compounds. Now, considering the nitrogen-based radicals (NH and NH_2_), they play a role in several reactions involved in the nitrogen chemistry of Titan. Among other reactions, NH_2_ is involved in the N_2_ production mechanism, which begins with the photodissociation of ammonia (NH_3_) into NH_2_ + H, and presents hydrazine (N_2_H_4_) and N_2_H_3_ as intermediates. Finally, NH is involved in recovering N_2_ to the atmosphere through reactions between the radicals NH + NH or NH + N, producing N_2_ + H_2_ and N_2_ + H, respectively. Hence, both radicals are also available to make part of the reactions to produce more complex molecules.

This work aims to check whether [Disp-formula eq3] and 4 are feasible reactions to produce CH_3_NH_2_ in such environments. The reactions were studied using electronic structure methods, variational transition state theory, and capture theory to obtain thermochemical and kinetic properties considering the conditions relevant to Titan’s atmosphere. These reactions were not considered by Krasnopolsky, which makes them an interesting line of study to contribute to the modeling of Titan’s atmosphere and the detection of nitrogen compounds off Earth.

## 2 Methodology

The electronic structure calculations were performed using the ORCA 5.0 package.^45,46^ The geometries of all stationary points (reactants, saddle points, products, and complexes) were properly optimized using the MP2 method with the cc-pVTZ basis set,^47,48^ as well as the ωB97X^49^ and ωB97X-D3 ^50^ methods, both employing the def2-TZVP basis set,^51^ and validated by the analysis of the harmonic frequencies, which were employed to calculate the zero-point energy (ZPE) for each stationary point. To ensure that the saddle points connect the reactants to the desired products, the intrinsic reaction coordinate (IRC) calculation^52,53^ was employed.

Gathering the results for these calculations, the obtained thermochemical properties were the electronic reaction energy (Δ*E*, the difference in electronic energy between products and reactants), the reaction enthalpy at 0 K (Δ*H*, Δ*E* + ΔZPE), the classical barrier height (*V* ^‡^, the difference in electronic energy between the saddle point and reactants), and the adiabatic barrier (Δ*V*_a_^*G*,‡^, *V* ^‡^ + ΔZPE^‡^). For the complexes along the reaction path, the obtained properties were Δ*E*_*c*_ (the difference in the electronic energy between the complex and the products) and Δ*H*_c_ (Δ*E*_c_ + ΔZPE).

The reliability of the previous techniques was assessed by comparing the results with single-point calculations using the CCSD­(T)/CBS//MP2/cc-pVTZ and CCSD­(T)/CBS//ωB97X/def2-TZVP methodologies, in which the extrapolation toward the complete basis set (CBS) limit was performed using the cc-pVTZ and cc-pVQZ basis sets. The extrapolations were performed as implemented in the ORCA 5.0 package, that is, the extrapolation for the energy values obtained by the SCF (self-consistent field) was based on the format proposed by Zhong et al.,^54^ and the correlation energy extrapolation was calculated using the procedure suggested by Helgaker et al.^55^ Both extrapolations used the exponents proposed by Neese and Valeev.^56^

The Pilgrim package^57,58^ was employed for the chemical kinetics calculations of the elementary steps with a saddle point (*R*_a2_ and *R*_b2_), where the rate coefficients were obtained via transition state theory (TST)^59,60^ and the canonical variational theory (CVT),^61,62^ for the temperature range from 75 to 300 K. For the construction of the minimum energy path (*V*_mep_) and the adiabatic potential curve (*V*_a_^*G*^) for *R*_a2_ and *R*_b2_, the dual-level method was employed using the interpolated single-point energies (ISPE) approach.^63^ The ωB97X/def2-TZVP method was used to calculate the properties along the reaction path (low-level), and the electronic results provided by the CCSD­(T)/CBS//ωB97X/def2-TZVP approach were employed as the high-level approach to improve this surface. Thus, the stationary points along the reaction coordinate are characterized by enthalpy and Gibbs free energy values given by the sum of the electronic energies obtained at CCSD­(T)/CBS with the zero-point energy and thermal corrections obtained at the ωB97X/def2-TZVP level. It is important to note that the Pilgrim package allows the use of the high-level method to correct electronic energies of configurations along the *V*_mep_ other than the stable geometries, further refining the reaction path. In these calculations, 18 more configurations along the reaction path were selected for this procedure. In order to take into account the nonclassical effects along the reaction coordinate, the small curvature tunneling approach (SCT)^61^ was employed.

The reactions *R*_*a*1_ and *R*_b1_ exhibited a direct stabilization of the energy from the reactants to the products without going through a saddle point. Thus, we applied the capture theory to estimate the rate coefficients for these steps.^64–66^ The theory applied to neutral bimolecular reactions considers dispersion forces as the main intermolecular interaction. The expression for the rate coefficients is given by

1 $$k\left(T\right)=\pi {\left(\frac{8k_{b}T}{\pi \mu }\right)}^{1/2}{\left(\frac{2C}{k_{b}T}\right)}^{1/3}\Gamma \left(\frac{2}{3}\right)$$

where Γ­(*x*) represents the Gamma function, μ is the reduced mass of the system, and *k*_*b*_ is the Boltzmann constant. *C* is known as the dispersion coefficient, and its value was estimated through a fitting of the potential energy surface calculated for the expression $V_{\mathrm{mep}}=-\frac{C}{R^{6}}$ , where *R* is the distance between the centers of mass of the molecules involved in the reaction. This theory is commonly used to obtain the rate coefficients for reactions occurring in the interstellar medium, and its applicability is limited to ultralow temperatures (around 5 K) up to room temperature (around 300 K).

For these steps that did not present a saddle point (*R*_a1_ and *R*_b1_), the minimum energy path was obtained using the relaxed scan and nudged elastic band (NEB) methodologies,^67^ respectively. For the relaxed scan calculation (*R*_a1_), we took the CH_2_NH_2_ structure optimized with ωB97X/def2-TZVP, increased the C–N bond length to 7.389 Å, and scanned this bond length until 1.389 Å in 600 steps, so that the last structure matches the optimized structure. For the NEB calculation (*R*_b1_), the supermolecule for the reactants CH_3_ + NH was optimized with ωB97X/def2-TZVP and used as the first image of the NEB calculations. This image was connected to the CH_3_NH optimized structure (last image) by employing 500 intermediate images.

## 3 Results and Discussion

For the methylamine production reaction, two reaction pathways, Ra and Rb, were studied, each consisting of two elementary steps. The first steps, *R*_a1_ and *R*_b1_, lead to the formation of the compounds CH_2_NH_2_ and CH_3_NH and the second steps, *R*_a2_ and *R*_b2_, yield CH_3_NH_2_ in its ground state and the nitrogen atom. The dehydrogenation reaction of methylamine assisted by the nitrogen atom was also studied through the two reaction pathways *R*_a3_ and *R*_b3_. Initially, MP2/cc-pVTZ, ωB97X/def2-TZVP, and ωB97X-D3/def2-TZVP methods were employed. The stationary points involved in these reactions, optimized and identified through harmonic frequency analysis, are illustrated in Figure 1, listing selected bond lengths, and the harmonic frequencies are listed in Table S1 in the Supporting Information. The intrinsic reaction coordinate (IRC) calculation was performed to verify the connection between reactants, products, and saddle points. This investigation led to the characterization of the CH_2_NH_2_···NH and CH_3_NH···NH complexes between the transition states and the reactants for *R*_a2_ and *R*_b2_, respectively.

**Figure 1:**
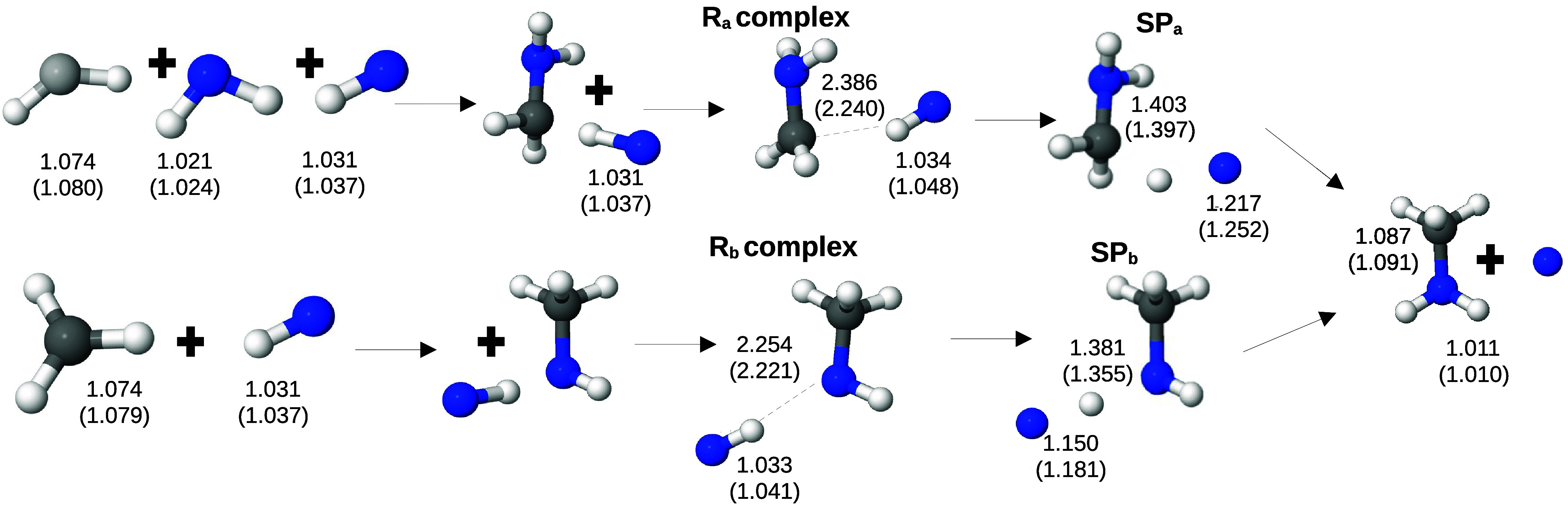
Reaction pathway for [Disp-formula eq3]: CH_2_ + NH_2_ + NH → CH_2_NH_2_ + NH → CH_3_NH_2_ + N, and 4: CH_3_ + 2NH → CH_3_NH + NH → CH_3_NH_2_ + N, with selected bond lengths (Å). The first values were obtained with the calculation level MP2/cc-pVTZ and in parentheses with ωB97X/def2-TZVP.

According to Hammond’s postulate,^68^ reactions with the saddle point closer to the products usually present a high barrier and are consequently endothermic, whereas reactions with the saddle point closer to the reactants generally exhibit a low barrier and are exothermic. For this analysis, we consider the geometries obtained by using the ωB97X/def2-TZVP method (Figure 1). First, analyzing *R*_a2_, the N–H bond distance changes by 0.215 Å between the reactants and SP_a_, while the C–H bond distance changes by 0.306 Å from SP_a_ to the products. Thus, we can consider that the saddle point is closer to the reactants, suggesting an exothermic reaction. Next, analyzing *R*_b2_, the N–H bond that is broken between the reactants and the saddle point exhibits a variation of 0.144 Å between these two structures, while the N–H bond formed between SP_b_ and the products changes by 0.345 Å. Therefore, SP_b_ is closer to the reactants, indicating that this reaction should also be exothermic. Furthermore, considering the variation in the N–H bond distance being broken between the reactants and the saddle point, this variation is greater for *R*_a2_ (0.215 Å) than for *R*_b2_ (0.144 Å). Consequently, we expect the barrier for *R*_*b*2_ to be lower than that for *R*_a2_.

To obtain reliable values for thermochemical properties, single-point calculations were performed using the CCSD­(T)/CBS method, initially considering the geometry optimized with the MP2/cc-pVTZ method. We believe this approach yielded results within the chemical accuracy of 4.2 kJ·mol^–1^. To verify this, we compared the enthalpy values at 0 K (Δ*H*) calculated using this method, which were −57.4 and −85.6 kJ·mol^–1^ for *R*_a2_ and *R*_b2_, respectively, with the values reported in the *Active Thermochemical Tables* database,^69^ maintained by the *Argonne National Laboratory*, for the same property, which are −54.0 and −82.4 kJ·mol^–1^. The values deviate by only 3.4 and 3.2 kJ·mol^–1^ for *R*_a2_ and *R*_b2_, respectively. Thus, we use the values calculated using the CCSD­(T)/CBS approach as a reference. The electronic energy profile for the reactions 3 and 4, obtained by this approach, is represented in Figure 2. According to this method, the electronic energy difference between reactants and products is −73.5 kJ·mol^–1^ for *R*_a2_ and −104.3 kJ·mol^–1^ for *R*_b2_. Considering the complex energy relative to the separated molecules, they exhibit stabilization of −12.9 kJ·mol^–1^ for [Disp-formula eq3] and −16.7 kJ·mol^–1^ for [Disp-formula eq4].

**Figure 2:**
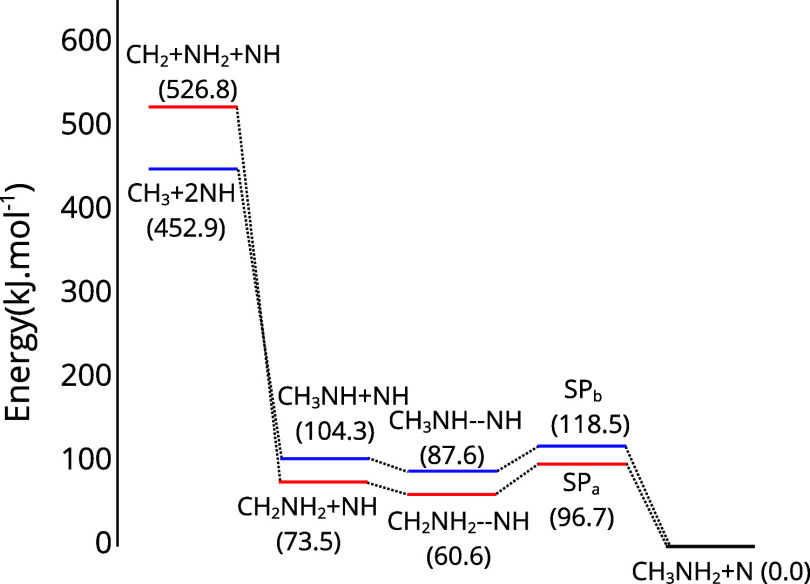
The electronic energy profile is shown in red for [Disp-formula eq3] and in blue for [Disp-formula eq4]. Energies were calculated with the CCSD­(T)/CBS//MP2/cc-pVTZ approach and are given in kJ·mol^–1^.

The thermochemical properties obtained using the MP2/cc-pVTZ, ωB97X/def2-TZVP, and ωB97X-D3/def2-TZVP methods are listed in Table 1.

**Table 1:** Thermochemical Data (kJ·mol^–1^) for Reaction Paths [Disp-formula eq3] and 4

[Disp-formula eq3]								
	Δ*E*_a1_	Δ*H*_a1_	Δ*E*_a2_	Δ*H*_a2_	*V* ^‡^	Δ*V*_a_^*G*,‡^	Δ*E*_c_	Δ*H*_c_
MP2/cc-pVTZ	457.8	421.6	–94.3	–78.2	26.9	30.9	–12.7	–6.6
ωB97X/def2-TZVP	466.5	430.3	–59.9	–42.8	17.2	19.8	–17.2	–10.6
ωB97X-D3/def2-TZVP	464.8	428.6	–60.2	–43.2	14.8	17.5	–16.2	–9.6
CCSD(T)/cc-pVTZ^a^	438.3	402.2	–85.1	–69.0	21.3	25.3	–12.7	–6.6
CCSD(T)/cc-pVQZ^a^	447.3	411.2	–77.7	–61.6	22.8	26.8	–12.6	–6.6
CCSD(T)/CBS^a^	453.3	417.2	–73.5	–57.4	23.2	27.2	–12.9	–6.8
CCSD(T)/cc-pVTZ^b^	437.7	401.6	–85.0	–67.8	20.8	23.4	–12.2	–5.6
CCSD(T)/cc-pVQZ^b^	447.0	410.8	–77.7	–60.5	22.4	25.1	–12.1	–5.5
CCSD(T)/CBS^b^	453.2	417.0	–73.5	–56.3	22.9	25.5	–12.3	–5.7

[Disp-formula eq4]								
	Δ*E*_b1_	Δ*H*_b1_	Δ*E*_b2_	Δ*H*_b2_	*V* ^‡^	Δ*V*_a_^*G*,‡^	Δ*E*_c_	Δ*H*_c_
MP2/cc-pVTZ	347.2	318.0	–132.5	–113.8	17.5	20.0	–18.3	–7.5
ωB97X/def2-TZVP	358.4	327.2	–89.2	–69.3	11.6	13.9	–20.1	–14.1
ωB97X-D3/def2-TZVP	356.8	325.8	–90.6	–70.6	9.1	11.6	–18.5	–11.5
CCSD(T)/cc-pVTZ^a^	333.2	304.0	–110.9	–92.3	12.3	14.9	–17.7	–7.0
CCSD(T)/cc-pVQZ^a^	468.9	439.7	–106.7	–88.1	13.9	16.4	–16.9	–6.1
CCSD(T)/CBS^a^	348.6	319.5	–104.3	–85.6	14.2	16.7	–16.7	–6.0
CCSD(T)/cc-pVTZ^b^	332.7	301.5	–110.6	–90.8	11.8	14.1	–17.6	–11.6
CCSD(T)/cc-pVQZ^b^	342.2	311.0	–106.6	–86.7	13.5	15.7	–16.7	–10.7
CCSD(T)/CBS^b^	348.5	317.3	–104.2	–84.4	13.8	16.1	–16.7	–10.6

a The geometry considered was optimized by the MP2/cc-pVTZ method.

b The geometry considered was optimized by the ωB97X/def2-tzvp method.

Regarding the barrierless steps, for *R*_a1_ we found the values of Δ*E*_a1_ to be 457.8 kJ·mol^–1^ (MP2), 466.5 kJ·mol^–1^ (ωB97X), and 464.8 kJ·mol^–1^ (ωB97X-D3), differing by a maximum of 8.7 kJ·mol^–1^. The values for Δ*H*_a1_ were also calculated, yielding 421.6, 430.3, and 428.6 kJ·mol^–1^, with a maximum difference of 8.7 kJ·mol^–1^. Using the CCSD­(T)/CBS//MP2/cc-pVTZ approach, the calculated values for Δ*E*_a1_ were 453.3 kJ·mol^–1^, and Δ*H*_a1_ was 417.2 kJ·mol^–1^. Thus, considering the CCSD­(T)/CBS calculations as a reference, the MP2 methodology yielded the best result for Δ*E*_a1_ (457.8 kJ·mol^–1^). For elementary step *R*_b1_, the values found for Δ*E*_*b*1_ were 347.2 kJ·mol^–1^ (MP2), 358.4 kJ·mol^–1^ (ωB97X), and 356.8 (ωB97X-D3) kJ·mol^–1^. For the CCSD­(T)/CBS//MP2/cc-pVTZ approach, the calculated Δ*E*_b1_ was 348.6 kJ·mol^–1^, and the MP2 methodology yielded the best result for Δ*E* (347.2 kJ·mol^–1^).

Now, considering the electronic barrier for *R*_a2_, our reference result yielded a value of 23.2 kJ·mol^–1^. The methodology that produced the closest result was MP2 (26.9 kJ·mol^–1^), followed by ωB97X (17.2 kJ·mol^–1^) and ωB97X-D3 (14.8 kJ·mol^–1^). For *R*_b2_, the reference value obtained was 14.2 kJ·mol^–1^, with the closest result given by the ωB97X functional (11.6 kJ·mol^–1^), followed by the MP2 (17.5 kJ·mol^–1^) and ωB97X-D3 (9.1 kJ·mol^–1^) methods. For Δ*E*, the reference value is −73.5 kJ·mol^–1^ for *R*_a2_, with the closest result obtained using the ωB97X-D3 functional (−60.2 kJ·mol^–1^). However, the ωB97X functional also yielded a very similar value (−59.9 kJ·mol^–1^), only 0.3 kJ·mol^–1^ from the value obtained with ωB97X-D3. The MP2 method produced the most divergent result with a value of −94.3 kJ·mol^–1^. For reaction pathway *R*_b2_, the reference value is −104.3 kJ·mol^–1^, with the closest result obtained using the ωB97X-D3 functional (−90.6 kJ·mol^–1^). Similarly to *R*_a2_, the value obtained with the ωB97X functional was also very close (−89.2 kJ·mol^–1^), showing a difference of only 1.4 kJ·mol^–1^ between them. The MP2 method again yielded the most distant result from the reference, with a value of −132.5 kJ·mol^–1^, corresponding to a deviation of 28.2 kJ·mol^–1^.

From this data set, we determined that the ωB97X/def2-TZVP method provided the most accurate values compared to the results obtained from single-point calculations using the CCSD­(T)/CBS//MP2/cc-pVTZ method. Therefore, ωB97X was used for the subsequent chemical kinetics calculations. In this context, we also considered the geometry optimized with the ωB97X functional for single-point calculations using the CCSD­(T)/CBS method, and the results are listed in Table 1. Comparing both sets of results obtained with the CCSD­(T)/CBS approach, the values differ by no more than 4.6 kJ·mol^–1^ (Δ*H*_c_ of [Disp-formula eq4]).

The minimum energy path for *R*_a1_ was estimated by using a relaxed scan calculation, which allows for the identification of the minimum energy path by varying the internal coordinates of the molecule. Thus, we selected the variation of the distance between the carbon and nitrogen atoms (see Figure 3 panel (a)) from 7.389 to 1.389 Å in 600 points, while keeping the dihedral angles H3–C1–N2–H6 and H4–C1–N2–H5 frozen and equal to −38.3 and 38.3°, as well as the bond angles between atoms C1–N2–H6 and C1–N2–H5 equal to 115.7°, and the angles N2–C1–H3 and N2–C1–H4 equal to 116.8°. For *R*_b1_, the surface was estimated by using the Nudged Elastic Band methodology, which also allows for the identification of the minimum energy path between the initial and final structures. To achieve this, a supermolecule calculation was performed for the CH_3_ + NH molecules, and this structure was connected to the CH_3_NH molecule, considering 500 images for this calculation. Both minimum energy paths were computed using the ωB97X/def2-TZVP methodology, and the resulting *V*_mep_ is found in Figure 3 panels (a) and (b).

**Figure 3:**
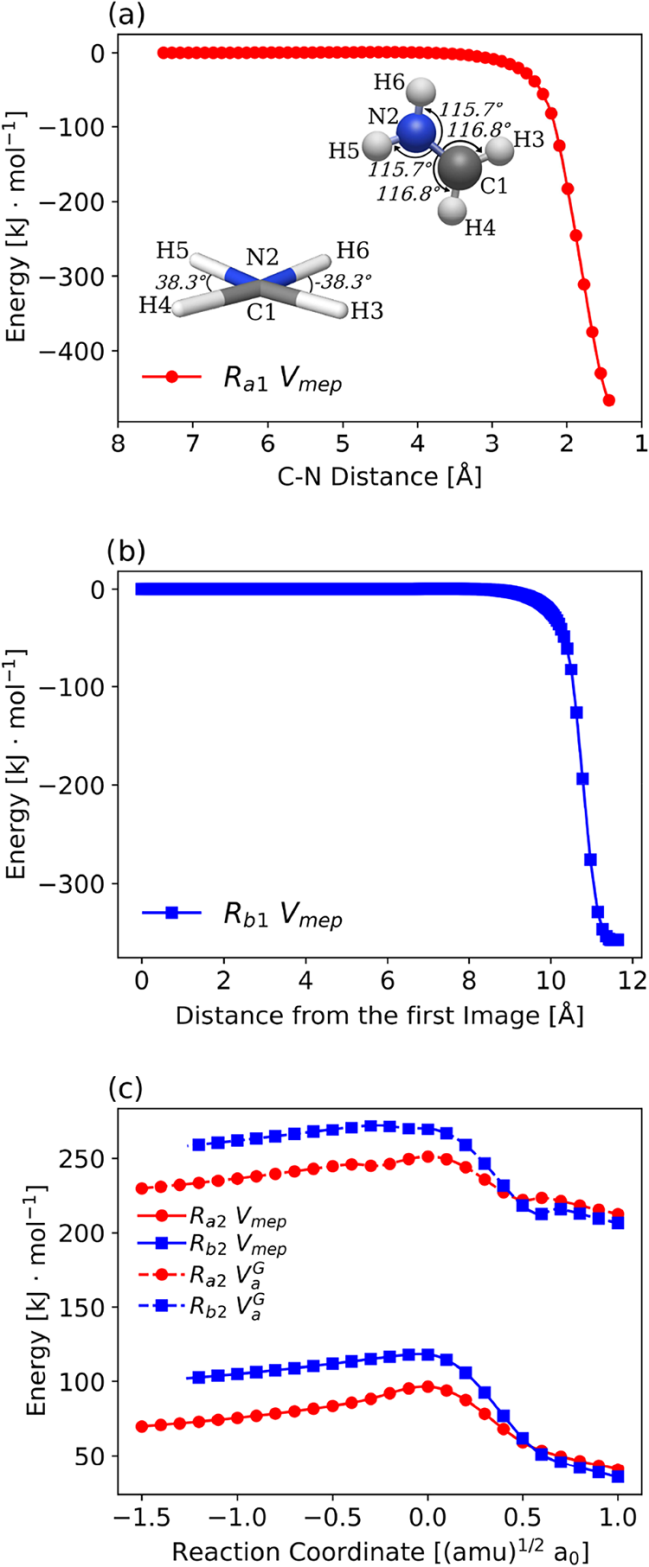
(a) Relaxed Scan starting at CH_2_ + NH_2_ obtained with ωB97X/def2-TZVP, highlighting the angles and dihedral angles frozen for the calculation. (b) NEB starting at CH_3_ + NH supermolecule connecting to the CH_3_NH optimized geometry obtained with ωB97X/def2-TZVP. (c) Electronic potential energy curves *V*_mep_ and adiabatic *V*_a_^*G*^ for the reactions *R*_a2_ in red and *R*_b2_ in blue, obtained by the dual-level methodology considering the ωB97X/def2-TZVP method as low-level and CCSD­(T)/CBS//ωB97X/def2-TZVP as high-level.

To ensure that the capture theory is suitable for the rate coefficient calculation, we calculated the Gibbs free energy curve at 100 K (in agreement with Titan’s atmosphere), obtaining the proper thermal and entropic contributions for all structures along the *V*_mep_. The plots are found in the SI (Figure S1). It can be seen that there is no barrier formed at the inner regions where the reaction takes place. In this context, the main barrier to be overcome is the centrifugal barrier that happens at long range, making capture theory suitable for these reactions.

The CVT method requires the calculation of *V*_mep_ and *V*_a_^*G*^. The *V*_mep_ is a path on the electronic potential energy surface. This surface considers the kinetic energy of electrons, Coulombic interactions between electrons, electrons and nuclei, and between nuclei. To account for the nuclear kinetic energy, the quantum harmonic oscillator approximation was employed. This allows us to calculate zero-point energies (ZPE) and harmonic frequencies. The *V*_a_^*G*^ curve is then obtained by adding the ZPE to the electronic energy of each point on the *V*_mep_.

The potential energy curves, *V*_mep_ and *V*_a_^*G*^, for *R*_a2_ and *R*_b2_ were obtained using the *dual-level* procedure, specifically the ISPE method (Interpolated Single-Point Energies).^63^ This approach combines two methodologies: (i) a computationally less expensive (low-level) method used to obtain thermal contributions and information along the reaction pathway and (ii) a high-level methodology to improve the electronic energies used to calculate the thermochemical properties along the path initially obtained by the low-level method. As aforementioned, the ωB97X/def2-TZVP approach was chosen as the low-level method, and the high-level method was selected as CCSD­(T)/CBS, using single-point calculations and considering the optimized geometry obtained with the same low-level method.

To apply the dual-level approach and improve the *V*_mep_, the electronic energy results from the high-level method are typically used only for stationary points (reactants, products, and saddle points). However, Pilgrim allows incorporating high-level information from additional points along the reaction path, enabling a better approximation of a surface calculated solely with the high-level method. Thus, in addition to the stationary points, high-level data were employed for 18 additional points along the reaction path. Indeed, it is crucial to acquire close agreement between the thermochemical properties obtained from low-level and high-level methodologies. Discrepancies between these two levels of theory can introduce artificial tunneling effects into the calculations, which in turn can contaminate the values of the rate coefficients.^70^ The vibrational modes of reactions *R*_a2_ and *R*_b2_ were projected onto internal coordinates, as implemented in the Pilgrim package. The resulting reaction pathways (*V*_mep_ and *V*_a_^*G*^) are shown in Figure 3 panel (c).

The capture theory was applied to estimate the rate coefficients for *R*_a1_ and *R*_b1_. For this purpose, we used the *V*_mep_ curves acquired for these reactions (Figure 3 panels (a) and (b)), which were fitted to an attractive potential of the form $-\frac{C}{R^{6}}$ , resulting in values of *C* = 9.4 × 10^3^ kJ·mol^–1^·Å ^6^ for *R*_a1_ and *C* = 11.5 × 10^3^ kJ·mol^–1^·Å ^6^ for *R*_b1_. This result allows us to report the rate coefficients for reactions *R*_a1_ and *R*_b1_ calculated employing eq 5 and are presented in Tables 2 and 3.

**Table 2:** Kinetic Data for Reaction Pathway [Disp-formula eq3] in cm^3^·Molecule^–1^·s^–1^, Obtained by the CCSD­(T)/CBS//*ω*B97X/def2-TZVP Approach

*R*_a1_	
*T*(K)	*k*_cap_
75	3.4 × 10^–9^
100	3.6 × 10^–9^
150	3.8 × 10^–9^
200	4.0 × 10^–9^
250	4.1 × 10^–9^
298.15	4.3 × 10^–9^
300	4.3 × 10^–9^

*R*_a2_			
*T*(K)	*k*_TST_	*k*_CVT_	*k*_CVT/SCT_
75	1.8 × 10^–30^	1.8 × 10^–30^	2.6 × 10^–18^
100	3.5 × 10^–26^	3.5 × 10^–26^	2.5 × 10^–18^
150	6.7 × 10^–22^	6.7 × 10^–22^	4.5 × 10^–18^
200	9.3 × 10^–20^	9.3 × 10^–20^	1.5 × 10^–17^
298.15	1.2 × 10^–17^	1.2 × 10^–17^	1.3 × 10^–16^
300	1.3 × 10^–17^	1.3 × 10^–17^	1.4 × 10^–16^

**Table 3:** Rate Coefficients for the Reaction Path [Disp-formula eq4] in cm^3^·molecule^–1^·s^–1^, Obtained by the CCSD­(T)/CBS//*ω*B97X/def2-TZVP Approach

*R*_b1_	
*T*(K)	*k*_cap_
75	3.6 × 10^–9^
100	3.8 × 10^–9^
150	4.1 × 10^–9^
200	4.3 × 10^–9^
250	4.4 × 10^–9^
298.15	4.6 × 10^–9^
300	4.6 × 10^–9^

*R*_b2_			
*T*(K)	*k*_TST_	*k*_CVT_	*k*_CVT/SCT_
75	5.4 × 10^–24^	1.1 × 10^–25^	7.0 × 10^–17^
100	2.5 × 10^–21^	1.4 × 10^–22^	6.5 × 10^–17^
150	1.1 × 10^–18^	1.8 × 10^–19^	9.4 × 10^–17^
200	2.4 × 10^–17^	6.4 × 10^–18^	2.2 × 10^–16^
298.15	4.9 × 10^–16^	2.2 × 10^–16^	1.1 × 10^–15^
300.0	5.1 × 10^–16^	2.3 × 10^–16^	1.1 × 10^–15^

For both reaction paths, there was no significant variation in the rate coefficients in the temperature range of 75–300 K, all being on the order of 10^–9^ cm^3^·molecule^–1^·s^–1^. The ratio of the rate coefficients at 300 and 75 K is 1.26 for *R*_a1_ and 1.28 for *R*_b1_; that is, the rate coefficient values vary by less than 30% in this temperature range.

These high values may be explained by two reasons. First, both are association reactions between radicals that lead to large energy release (430.3 kJ·mol^–1^ for *R*_a1_ and 327.2 kJ·mol^–1^ for *R*_b1_), that is, the reactivity of these radicals favors the reactions. Second, these rate coefficients are valid for the high-pressure limit, i.e., the rate coefficient is determined solely by the formation of the products CH_2_NH_2_ and CH_3_NH, since these products are collisionally stabilized by the inert body M. Otherwise, the products might retain enough energy to redissociate into reactants. This approximation is adequate for Titan’s atmosphere, especially for the lower atmosphere, since this environment presents a high molecular density, and the concentration of M can be approximated to the total concentration of colliders, providing a sufficiently high collision rate to stabilize the products and achieve the high rate coefficients for collision-stabilized radical-radical associations.^44^

The forward (*k*_a2_ and *k*_b2_) rate coefficients for both reactions *R*_a2_ and *R*_b2_ are also presented in Tables 2 and 3. The reverse rate coefficients (*k*_a3_ and *k*_b3_) are found in the Supporting Information at Tables S2 and S3. These values were obtained using the TST, CVT, and CVT/SCT methods over a temperature range of 75–300 K. We selected this temperature range primarily because our main interest lies in Titan’s atmosphere (∼100 K).^11–14^ However, this range can also represent conditions relevant to molecular clouds (50–300 K),^3^ nebulae (∼70 K),^4,5^ and the atmospheres of Jovian planets (100–200 K).^9^ Additionally, we performed calculations over a broader temperature range (up to 2000 K), with the corresponding results also provided in the Supporting Information in Tables S2 and S3. These extended values may be applicable to other environments such as rocky exoplanets (1000–1500 K).^15^ The results from 75 to 300 K are also presented as Arrhenius plots of  *k* vs 1/*T* (Figure 4). In the TST methodology, the energy barrier that the reactants must overcome is given by the difference in Gibbs free energy at each temperature between the saddle point (considered the transition state) and the reactants (Δ*G* ^‡^). The CVT method is employed to improve this approximation by incorporating variational effects that identify the maxima on the Gibbs free energy surface along the reaction path, providing a more accurate representation of the transition state.

**Figure 4:**
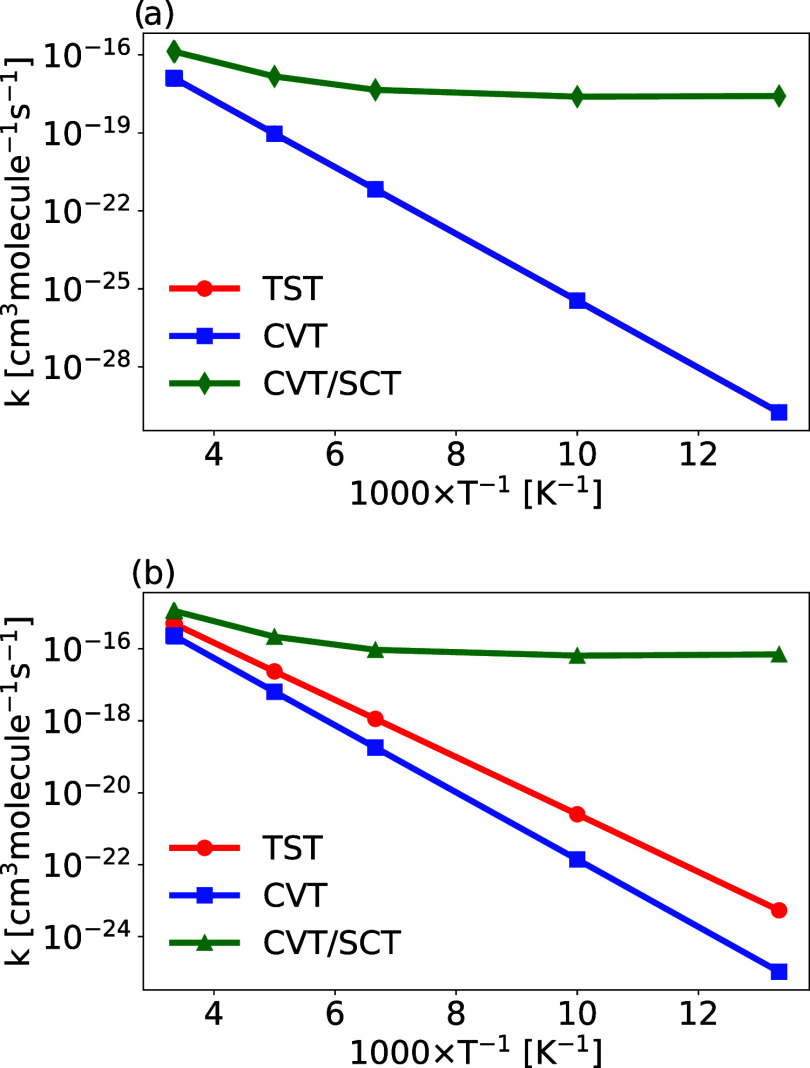
Arrhenius plot (*k* × 1000·*T* ^–1^) for reactions *R*_a2_ (panel­(a)) and *R*_b2_ (panel (b)).

By considering the variational effect, which can be analyzed through the ratio of rate coefficients obtained from CVT and TST methods, it is observed that at 100 K, this ratio is 1.00 for *R*_a2_, indicating that the effect is negligible and the curves in Figure 4 panel (a) coincide. For *R*_b2_, the ratio is 0.06, and the plot shows the CVT curve lying below the TST curve (Figure 4 panel (b)). This effect becomes less significant at higher temperatures, with the ratio reaching 0.45 for *R*_b2_ at 300 K.

The quantum effects estimated by using the SCT approach were applied to the rate coefficients obtained via the CVT method to account for the possibility of reactants transforming into products even without possessing the thermal energy required to reach the transition state. The ratio of the rate coefficients obtained from the CVT/SCT and CVT methods at 100 K is 7.1 × 10^7^ for *R*_a2_ and 4.6 × 10^5^ for *R*_b2_. This analysis shows the importance of considering the tunneling effect to obtain reliable results. Thus, at 100 K, a temperature consistent with Titan’s atmosphere, our best values for the rate coefficients are equal to 2.5 × 10^–18^ and 6.5 × 10^–17^ cm^3^ · molecule^–1^·s^–1^ for *R*_a2_ and *R*_b2_, respectively.

## 4 Implications in Titan’s Atmosphere

This study was motivated by the atmospheric modeling of Titan conducted by Krasnopolsky,^11^ which reported the following reactions: CH_3_NH_2_ + *h*ν → CH_3_ + NH_2_ with a yield of 1.3 × 10^–6^ s^–1^ and NH_2_ + CH_3_ + M → CH_3_NH_2_ + M with a rate coefficient of 6 × 10^–18^ *T* ^–3.85^, 6 × 10^15^ cm^6^·s^–1^ (with *T* being the temperature, considering *T* = 170 K), where the first value describes the low pressure rate coefficient, and the second value is the high-pressure rate coefficient. In this study, we propose alternative reaction paths for the production and decomposition of methylamine ([Disp-formula eq3] and 4), considering atomic nitrogen, as it is an element present in Titan’s atmosphere.^71–73^

Based on the thermochemical and kinetic data of both reaction paths, it is evident that the formation of CH_3_NH_2_ + N is more favorable than its decomposition. For the reaction paths *R*_a1_ and *R*_b1_, the calculated rate coefficient at 100 K were 3.6 × 10^–9^ and 3.8 × 10^–9^ cm^3^·molecule^–1^·s^–1^, respectively. These addition reactions store 417.0 and 317.3 kJ·mol^–1^ in the respective products. In the context of Titan’s atmosphere, we consider that it is dense enough for a third inert body to thermalize and stabilize the products CH_2_NH_2_ and CH_3_NH. The products may react again with the NH radical, with the barriers to overcome equal to 25.5 kJ·mol^–1^ (*R*_a2_) and 16.1 kJ·mol^–1^ (*R*_b2_) for the production of methylamine. The rate coefficients found for the production of methylamine were 2.5 × 10^–18^ and 6.5 × 10^–17^ cm^3^·molecule^–1^·s^–1^, at 100 K, for *R*_a2_ and *R*_b2_, respectively. It is worth noting that, considering the conditions of Titan’s atmosphere, the rate coefficient of the elementary step *R*_b2_ is greater than that of *R*_a2_. The least likely case is for the third inert body to be the NH molecule, but in this case, the stored energy can be used to overcome these barriers, and the excess energy can be released in the two species that form the final product.

The rate coefficients obtained for its decomposition at 100 K (*R*_a3_ and *R*_b3_), were equal to 4.7 × 10^–47^ and 3.1 × 10^–60^ cm^3^·molecule^–1^·s^–1^, respectively, and At 300 K the values of the rate coefficients for the decomposition of methylamine found were: 2.9 × 10^–25^ and 3.9 × 10^–29^ cm^3^·molecule^–1^·s^–1^ for *R*_a3_ and *R*_b3_, respectively, that is, if the decomposition occurs, it is more favored at high temperatures.

Since Krasnopolsky’s mechanism^11^ only includes the pathway CH_3_ + NH_2_ → CH_3_NH_2_ ([Disp-formula eq2]) for the formation of this molecule, and its potential photodissociation ([Disp-formula eq1]), our results can be incorporated into such mechanisms to simulate that atmosphere more accurately. This inclusion offers a viable alternative route for the formation of methylamine in that environment from the radicals CH_3_ + NH and CH_2_ + NH_2_, which are likely to be present in Titan’s atmosphere.^44^

We believe that our findings could also encourage observational scientists to search for methylamine within the existing data for Titan’s atmosphere and other similar environments. This could potentially expand the understanding of the chemical processes occurring in Titan’s rich and complex atmospheric composition and possibly lead to the detection of methylamine, which may play a role in the chemistry of Titan, as well as in other similar environments.

Moreover, our results also suggest the production of CH_2_NH_2_ and CH_3_NH radicals. Given their presence in Titan’s atmosphere, they are also available to react with other molecules in that environment, like nitrogen compounds and hydrocarbons, unlocking other reaction paths and further improvements for modeling Titan’s atmosphere, which is crucial for understanding, for example, the presence of hydrocarbons or nitrogen compounds.

## 5 Conclusions

The aim of our work was to study the thermochemical and kinetic properties of the proposed reaction paths involving the production and decomposition of methylamine under conditions consistent with Titan’s atmosphere. It is worth mentioning that this molecule also serves as a precursor in the synthesis of proteinogenic amino acids. Thus, our results also contribute to a better understanding about the production of complex organic molecules in the interstellar medium. Some works, such as that of Krasnopolsky,^11^ simulate the atmosphere of Titan, where two elementary steps involving methylamine were considered, its photodissociation forming the radicals CH_3_ and NH_2_ and its production from these radicals in the presence of an inert body. In this study, we outline more reactions by which these processes can occur, given by the paths described in [Disp-formula eq3] and 4.

Concerning the thermochemical data, our best calculations were obtained with the CCSD­(T)/CBS//ωB97X/def2-TZVP methodology, and subsequent chemical kinetic calculations were carried out using the ωB97X/def2-TZVP approach to obtain the properties along the reaction paths. For *R*_a2_ and *R*_b2_, the minimum energy paths were improved by using the values obtained by the CCSD­(T)/CBS method. The capture theory was employed for the rate coefficient calculations of *R*_a1_ and *R*_b1_, while the CVT/SCT method was used to obtain the rate constants for *R*_a2_ and *R*_b2_.

[Disp-formula eq3] starts with the radicals CH_2_ and NH_2_ proceeding via a barrierless addition reaction to produce CH_2_NH_2_ (*R*_a1_), releasing 417.0 kJ·mol^–1^ and with a rate coefficient equal to 3.6 × 10^–9^ cm^3^·molecule^–1^·s^–1^ at 100 K. Given the availability of the NH radical and enough energy to overcome the barrier of 25.5 kJ·mol^–1^, the reaction can proceed to the second step *R*_a2_ leading to the formation of the products CH_3_NH_2_ and atomic nitrogen, releasing 73.5 kJ·mol^–1^ with a rate coefficient of 2.5 × 10^–18^ cm^3^·molecule^–1^·s^–1^. The other proposed path [Disp-formula eq4] starts with a barrierless addition reaction between the radicals CH_3_ and NH, forming CH_3_NH (*R*_b1_), releasing 317.3 kJ·mol^–1^ with a rate constant of 3.8 × 10^–9^ cm^3^·molecule^–1^·s^–1^. The barrier to overcome in the following step *R*_b2_ is equal to 16.1 kJ·mol^–1^ and also leads to the formation of methylamine and atomic nitrogen with a rate constant equal to 6.5 × 10^–17^ cm^3^·molecule^–1^·s^–1^. In the first steps of the reactions, the presence of a third inert body is necessary to stabilize the product species and dissipate energy in order to avoid the dissociation via the reverse path. The study was also carried out for the reverse processes, the decomposition of methylamine indicated by steps *R*_a__3_ and *R*_b3_. These elementary reactions are endothermic, and the rate constants are lower than 10^–46^ cm^3^ ·molecule^–1^·s^–1^, indicating that the production of methylamine is more favorable than its decomposition for the reactions considered.

Within the context of Titan, the initial chemical species proposed in the reaction pathways are present in its atmosphere,^44^ indicating the viability of the reactions 3 and 4. Moreover, these reactions may be included to further improve the mechanisms to simulate the atmosphere of Titan, enhancing our knowledge of that moon. Moreover, our results may also be employed in other dense environments, serving to identify areas where methylamine can be detected outside of Earth.

## Supplementary Material



## Notes

Harmonic frequencies for all stationary structures; *V*_mep_, *V*_a_^*G*^, and free Gibbs energy at 100 K for *R*_a1_ and *R*_b1_; additional rate coefficients for [Disp-formula eq3] and 4; Complete set of xyz coordinates for the stable structures (PDF)
