# Supplementary material for: Thermochemical and Kinetic Investigation of CH3NH2 Production in Titan’s Atmosphere
Source: ACS Omega. 2025 Nov 17;10(46):56597–606. doi: 10.1021/acsomega.5c09060 (PMC12658629; doi:10.1021/acsomega.5c09060)
Supplement: Supplementary file 1 [file ao5c09060_si_001.pdf]

Thermochemical and Kinetic Investigation of CH_3NH_2 Production in Titan's Atmosphere

Ghuerda L. Mayr[1], Isabela S. Vieira[1], and Rene F. K. Spada[1,2]

[1]Departamento de Física, Instituto Tecnológico de Aeronáutica, São José dos Campos, São Paulo, 12.228-900, Brasil
[2]Laboratório de Computação Científica Avançada e Modelamento (Lab-CCAM), Instituto Tecnológico de Aeronáutica, São José dos Campos 12228-900, SP, Brazil

Supplementary Information

Table S1: Harmonic Frequencies in cm^{-1}calculated in ωB97X/def2-TZVP.

CH_2	CH_3	NH_2	CH_3NH_2	SP_a	CH_2NH_2	NH	SP_b	CH_3NH
1069	525	1530	277	1822i	433	3323	1825i	263
3155	1406	3416	818	127	588		140	955
3384	1409	3511	973	264	657		147	1011
	3142		1097	493	936		507	1089
	3322		1172	522	1251		724	1337
	3323		1348	700	1325		983	1401
			1464	968	1488		1011	1484
			1504	1067	1661		1066	1487
			1518	1184	3181		1091	3001
			1671	1194	3293		1190	3050
			3017	1252	3602		1352	3140
			3105	1354	3702		1434	3453
			3144	1480			1478	
			3561	1673			1507	
			3640	3113			3031	
				3202			3091	
				3595			3149	
				3688			3474	

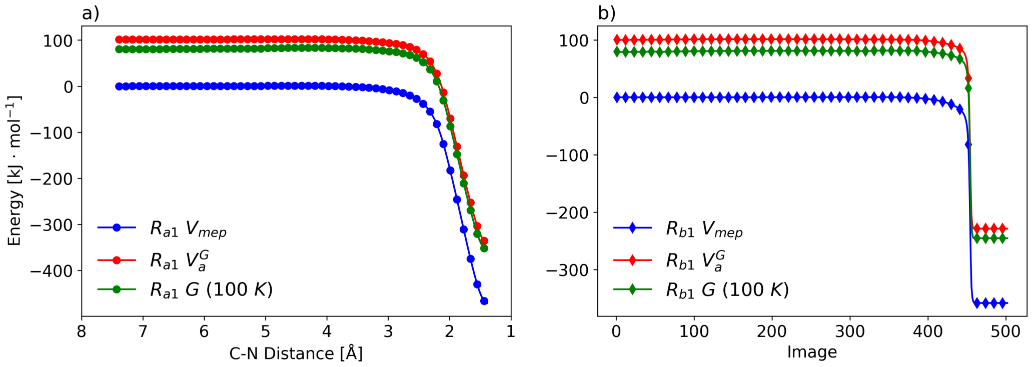

Figure S1: V_{mep}, V_a^G and free Gibbs energy at 100 K for R_{a1} (panel (a)) and R_{b1} (panel (b)).

Table S2: Rate coefficients for the reaction path R_a in $cm^3 \cdot molecule^{-1} \cdot s^{-1}$.

	Ra_2		
T(K)	k_{TST}	k_{CVT}	$k_{CVT/SCT}$
400.0	1.6×10^{-16}	1.6×10^{-16}	6.4×10^{-16}
500.0	7.9×10^{-16}	7.9×10^{-16}	1.9×10^{-15}
700.0	5.6×10^{-15}	5.6×10^{-15}	8.9×10^{-15}
1000.0	3.0×10^{-14}	3.0×10^{-14}	3.8×10^{-14}
1500.0	1.6×10^{-13}	1.6×10^{-13}	1.8×10^{-13}
2000.0	4.7×10^{-13}	4.7×10^{-13}	5.0×10^{-13}
	Ra_3		
T(K)	k_{TST}	k_{CVT}	$k_{CVT/SCT}$
75.0	4.1×10^{-69}	4.1×10^{-69}	6.1×10^{-57}
100.0	6.8×10^{-55}	6.8×10^{-55}	4.7×10^{-47}
150.0	1.2×10^{-40}	1.2×10^{-40}	7.9×10^{-37}
200.0	1.7×10^{-33}	1.7×10^{-33}	2.7×10^{-31}
298.15	2.2×10^{-26}	2.2×10^{-26}	2.4×10^{-25}
300.0	2.8×10^{-26}	2.8×10^{-26}	2.9×10^{-25}
400.0	1.3×10^{-22}	1.3×10^{-22}	5.2×10^{-22}
500.0	2.4×10^{-20}	2.4×10^{-20}	5.9×10^{-20}
700.0	1.1×10^{-17}	1.1×10^{-17}	1.8×10^{-17}
1000.0	1.5×10^{-15}	1.5×10^{-15}	1.9×10^{-15}
1500.0	8.8×10^{-14}	8.8×10^{-14}	9.8×10^{-14}
2000.0	8.1×10^{-13}	8.1×10^{-13}	8.6×10^{-13}

Table S3: Rate coefficients for the reaction path R_b in $cm^3 \cdot molecule^{-1} \cdot s^{-1}$.

	Rb_2		
T(K)	k_{TST}	k_{CVT}	$k_{CVT/SCT}$
400.0	2.5×10^{-15}	1.5×10^{-15}	3.7×10^{-15}
500.0	7.0×10^{-15}	4.8×10^{-15}	8.7×10^{-15}
700.0	2.7×10^{-14}	2.1×10^{-14}	2.8×10^{-14}
1000.0	9.3×10^{-14}	7.9×10^{-14}	9.0×10^{-14}
1500.0	3.5×10^{-13}	3.0×10^{-13}	3.2×10^{-13}
2000.0	8.7×10^{-13}	7.5×10^{-13}	7.6×10^{-13}
	Rb_3		
T(K)	k_{TST}	k_{CVT}	$k_{CVT/SCT}$
75.0	4.0×10^{-82}	7.8×10^{-84}	5.2×10^{-75}
100.0	1.2×10^{-64}	6.7×10^{-66}	3.1×10^{-60}
150.0	3.9×10^{-47}	6.3×10^{-48}	3.3×10^{-45}
200.0	2.4×10^{-38}	6.6×10^{-39}	2.2×10^{-37}
298.15	1.3×10^{-29}	6.1×10^{-30}	3.1×10^{-29}
300.0	1.7×10^{-29}	7.9×10^{-30}	3.9×10^{-29}
400.0	5.2×10^{-25}	3.1×10^{-25}	7.7×10^{-25}
500.0	2.8×10^{-22}	1.9×10^{-22}	3.5×10^{-22}
700.0	4.5×10^{-19}	3.5×10^{-19}	4.7×10^{-19}
1000.0	1.4×10^{-16}	1.2×10^{-16}	1.4×10^{-16}
1500.0	1.8×10^{-14}	1.5×10^{-14}	1.6×10^{-14}
2000.0	2.3×10^{-13}	2.0×10^{-13}	2.1×10^{-13}

Methylamine (CH_3NH_2) coordinates (Å)
Optimization: ωB97x/def2-TZVP Energy = -95.86784857 Eh
CCSD(T)/CBS//ωB97X/def2-TZVP Energy = -95.746823330341 Eh

C	0.049518	0.701290	0.000100
N	0.050786	-0.754816	0.000176
H	-0.946296	1.163467	0.000027
H	0.587264	1.063329	0.877741
H	0.587356	1.063250	-0.877521
H	-0.427587	-1.115949	-0.812940
H	-0.427442	-1.115871	0.813415

CH_2NH_2 coordinates (Å).
Optimization: ωB97x/def2-TZVP Energy = -95.21112042 Eh
CCSD(T)/CBS//ω97X/def2-TZVP Energy = -95.086762832681 Eh

C	0.206748	0.636475	0.000136
N	-0.003109	-0.736286	0.000200
H	0.530355	1.080930	0.930822
H	0.530302	1.080847	-0.930608
H	-0.420868	-1.114565	-0.833647
H	-0.420826	-1.114501	0.834096

CH_3NH coordinates (Å).
Optimization: ωB97x/def2-TZVP Energy = -95.19998031 Eh
CCSD(T)/CBS//ωB97X/def2-TZVP Energy = -95.075061384384 Eh

C	0.073538	0.685088	0.006097
N	0.047287	-0.747203	0.024654
H	-0.942675	1.102560	-0.023429
H	0.585768	1.073121	0.886463
H	0.581802	1.062021	-0.892594
H	-0.446820	-1.049787	-0.817891

CH_3 coordinates (Å).
Optimization: ωB97x/def2-TZVP Energy = -39.83796984 Eh
CCSD(T)/CBS//ωB97X/def2-TZVP Energy = -39.779462884412 Eh

C	-0.000000	-0.000033	-0.000000
H	0.000000	1.079425	0.000000
H	0.934851	-0.539696	0.000000
H	-0.934851	-0.539696	0.000000

CH_2 coordinates (Å).
Optimization: ωB97x/def2-TZVP Energy = -39.15077883 Eh
CCSD(T)/CBS//ωB97X/def2-TZVP Energy = -39.093037492002 Eh

C	0.000000	0.000000	0.093494
H	0.000000	0.996250	-0.322747
H	0.000000	-0.996250	-0.322747

NH_2 coordinates (Å).
Optimization: ωB97x/def2-TZVP Energy = -55.88267715 Eh
CCSD(T)/CBS//ωB97X/def2-TZVP Energy = -55.821123049556 Eh

N	0.000000	0.000000	0.140075
H	0.000000	0.805618	-0.492850
H	0.000000	-0.805618	-0.492850

NH coordinates (Å).
Optimization: ωB97x/def2-TZVP Energy = -55.22549569 Eh
CCSD(T)/CBS//ωB97X/def2-TZVP Energy = -55.162860961143 Eh

N	0.000000	0.000000	0.181257
H	0.000000	0.000000	1.218743
